# Human metapneumovirus (hMPV): an associated etiology of severe acute respiratory infection in children of Eastern Uttar Pradesh, India

**DOI:** 10.1099/acmi.0.000829.v4

**Published:** 2024-09-12

**Authors:** Hirawati Deval, Niraj Kumar, Mitali Srivastava, Varsha Potdar, Anita Mehta, Ayushi Verma, Rajeev Singh, Asif Kavathekar, Rajni Kant, Manoj Murhekar

**Affiliations:** 1 ICMR- Regional Medical Research Centre, Gorakhpur, India; 2 National Influenza Centre, ICMR- National Institute of Virology, Pune, India; 3 Department of Paediatrics, BRD Medical College, Gorakhpur, India; 4 ICMR- National Institute of Epidemiology, Chennai, India

**Keywords:** acute respiratory infection, human metapneumovirus, inpatient department, outpatient department, severe acute respiratory infection

## Abstract

Acute respiratory infections (ARIs) are a serious public health concern across the world, causing considerable morbidity and mortality. Every year, around 13 million children under the age of five die. Approximately 95% of them are from developing nations, and ARIs are responsible for one-third of all deaths. Human Metapneumovirus (hMPV) is one of the causative agents associated with respiratory tract infections. There is lack of information about hMPV from the eastern region of Uttar Pradesh. At Indian Council of Medical Research- Regional Medical Research Centre, Gorakhpur (ICMR‐RMRC, Gorakhpur) in Uttar Pradesh, India; we tested respiratory pathogens in under-five patients presenting with ARI and severe acute respiratory illness (SARI) through semi nested PCR. A total of 100 nasal and throat specimens were collected from the outdoor and indoor patient Departments (OPD) and (IPD) of Department of Paediatrics, BRD Medical College, Gorakhpur during February to April 2022. Out of 100 enrolled paediatric patients, 4(4%) were found to be hMPV positive. Among the patients who tested positive for hMPV, 25%(1/4) unfortunately died. The phylogenetic analysis of hMPV showed the close resemblance with the clade of Singapore and USA hMPV isolates. The study underlines the importance of hMPV as the cause of acute respiratory infections in children and the highlight the need for routine testing for this virus in laboratories. Further more comprehensive detailed study on various aspects of hMPV in this area is needed.

## Data Summary

All data related to this work as reported in this article such as the Matrix gene sequence of the PCR amplified product used in the phylogenetic analysis has been deposited in GenBank under accession no. OR453272/OR453273/OR463876/OR463878.

## Introduction

The Acute Respiratory Infection (ARI) results in an estimated 1.9 to 2.2 million deaths every year, of which 70% occur in developing countries which accounts for 30% of total childhood deaths [1,2]. Human metapneumovirus (hMPV) has been described as an important aetiologic agent of upper and lower respiratory tract infections, especially in children below 5 years of age and the elderly people. Most common symptoms of hMPV are fever, breathlessness, nasal congestion, rhinorrhea, cough, sore throat, and headache [3]. According to a study in Cambodia, hMPV infection was 0.2% in 2007, 4.3% in 2008 and 0.3% in 2009 [4]. According to another study, the estimated prevalence of hMPV infections among hospitalized ARI was 6.24% [5]. In Chennai, South India a study was conducted between April 2016 and August 2018 and 350 nasal swab specimens were obtained from the children with ARI and analysed for hMPV using the real-time PCR technique. In this investigation, hMPV was found in 4% of the samples (14/350) [6].

hMPV is a single-stranded negative-sense enveloped RNA virus under the family *Pneumoviridae*. The virus genome is 13.35 kb in length and encodes eight genes which encode nine proteins such as N (nucleoprotein), P (phosphoprotein), M (matrix protein), F (fusion protein), M2 (transcription enhancer protein), SH (small hydrophobic protein), G (attachment glycoprotein) and L (RNA dependent RNA polymerase) [7].

The matrix protein of hMPV is secreted by infected cells in a soluble form and induces the secretion of inflammatory cytokines [8]. This protein is a major component of the virus and aids in viral assembly and budding [9]. Based on the previous reports, hMPV infection appears to be seasonal, and co-infection with other respiratory pathogens is common [10]. However, there are still no vaccines or antivirals that can be used to treat hMPV infection. This study was aimed at molecular characterization of hMPV in children below 5 years of age in eastern UP. We have amplified matrix protein gene of hMPV through conventional RT-PCR, and then the sequencing and phylogenetic analysis for the product were done.

## Methods

### Sample collection

The patients were enrolled according to the WHO case definition of acute respiratory infection (ARI) and severe acute respiratory infection (SARI). Patients with fever (>38 °C), cough and with onset within the last 10 days were classified as ARI and patients with sudden onset of fever (>38 °C), breathlessness, cough, sore throat that required hospital admission categorized as SARI [11].

The nasal and throat swab samples were collected from the patients between the age group of 1–60 months from outpatient department (OPD) and inpatient department (IPD) of Paediatrics, BRD Medical College, Gorakhpur, Uttar Pradesh.

### Molecular detection

The nucleic acid extraction was done using QIAGEN RNA mini kit (Qiagen, Hilden, Germany, Catalogue No.-52904) and RNA was eluted in 60 µl of elution buffer. The RNA was changed to complementary DNA by reverse transcription firstly, and then subjected to conventional semi nested PCR using primers (Table 1) based on matrix protein (M) gene of hMPV. The reverse transcription step was done using AMV RT kit (Promega; Catalogue no.- M5101). Reactions were performed with the following conditions: reverse transcription 45 °C 45 min; AMV RT inactivation and cDNA/primer denaturation at 94 °C for 5 min. For M-protein gene outer PCR, the conditions were: denaturation at 94 °C 30 s, annealing at 58 °C 45 s, extension at 72 °C 1 min (35 cycles), and final extension 72 °C 7 min, 4 °C (hold). For semi-nested PCR, the conditions were: denaturation at 94 °C 5 min, 35 cycles of 94 °C 30 s, 58 °C 45 s, 68 °C 1 min, and final extension 68 °C 7 min, 4 °C (hold).

**Table 1.** Sequences of primers used for amplification of matrix protein coding gene of hMPV by PCR

Primers	Sequences
hMPV226F	ATA TGG TTC CCT TTG TTT CAG GC
hMPV685R	TGG TCT GCT TCA CTG CTT ATW GCA GCT T
hMPV664R	GCA GCT TCA ACA GTR GCT GAT TCA CTC TC

The viral RNA was also tested for routine diagnostics, for other respiratory panels (Influenza-A and B, SARS CoV-2, RSV-A and B, Parainfluenza virus-1, 2, 3, and 4, Adenovirus, Human Rhinovirus) by real time reverse transcription polymerase chain reaction (rRT-PCR) [12,14].

### Phylogenetic analysis

The PCR amplified product of 439 bp were gel purified and further sequenced by respective forward (hMPV226F) and reverse primer (hMPV664R) using Sanger Sequencing and run onto ABI Prism 3130 Genetic analyzer (Applied Biosystems, Foster City, CA, USA). The results obtained were analysed for the pairwise comparison with NCBI database (www.ncbi.nlm.nih.gov) with worldwide hMPV sequences by Basic Local Alignment Search Tool (blast) analysis programme (https://blast.ncbi.nlm.nih.gov/Blast.cgi). Multiple sequence alignment was performed using clustal W Programme. Phylogenetic tree was constructed on the basis of the Maximum Likelihood Method with 1000 bootstrap replicates and rooted to avian metapneumovirus using mega 11.0 software [15,16].

## Results

A total of 100 paediatric patients were enrolled as per the case definition, from February 2022 to April 2022. Out of the 100 patients we enrolled, 50 were of ARI and 50 were of SARI. The viral RNA was extracted and tested for routine diagnostics, as well as for other viral respiratory panels (Influenza-A and B, SARS CoV-2, RSV-A and B, Parainfluenza virus-1, 2, 3, and 4, Adenovirus, Human Rhinovirus) and DNA was extracted and tested for bacteria (*Haemophilus influenzae*, *Streptococcus pneumoniae*). All these aetiologies were found negative and the samples were further screened by the hMPV genus specific semi-nested RT-PCR. Out of 100 enrolled paediatric patients, 4(4%) were found to be positive. The clinical information for patients that tested negative for hMPV was not obtained from the hospital. The four positive cases were SARI patients, which constituted 8% of total SARI positive patients in our study and admitted in Intensive Care Unit (ICU), Department of Paediatrics, BRD Medical College, Gorakhpur, Uttar Pradesh, India. From the four positive patients, 2 were male and two were females. The median age of positive patients was 34 months. The hMPV positivity was found to be similar in the age group, with one case each in the age group of 0–12 months (1%), 13–24 months (1%), 37–48 (1%) and 49–60 months (1%). There was no positivity in the age group of 25–36 months. The clinical and biochemical findings of four hMPV positive patients are summarized in Table S1, available in the online version of this article.

### Patient 1

A 24-month-old male patient was admitted to the emergency ward with fever, cough, vomiting for four days and difficulty in breathing for one day. At physical examination, his pulse rate was found to be higher (164 per min) and oxygen saturation was low (90%). The patient was treated with Ceftriaxome, Ondem and Pantoprazole (as per routine treatment).The patient condition improved and he was discharged on satisfactory grounds after three days of hospital admission.

### Patient 2

A 60-month-old male patient was admitted to the emergency ward with fever, cough, vomiting and difficulty in breathing for 12 h. The patient was treated with Ondem, Pantoprazole and Atopine (as per routine treatment), and the patient condition improved and he was discharged after two days of hospital admission.

### Patient 3

A 5-month-old female patient was admitted to the emergency ward with fever, cough, vomiting and difficulty in breathing. She was known to have Cyanotic Congenital Heart Disease with Respiratory distress. At physical examination her Pulse Rate (PR) was found to be 98 per min, Respiratory Rate (RR) was 52 per minute and oxygen saturation was 86%. On examination, significant cyanosis was observed. Her four limbs saturation was RUL: 77%, LUL- 84%, RLL- 86%, LLL-71%. Patient was given Oxygen support via Nasal Prongs/Nasal Cannula for one day and was managed with antibiotic ceftriaxone for 2 days. Patient was referred to higher centre for further management.

### Patient 4

A 44-month-old female patient was admitted to the emergency ward because of fever, cough, vomiting and difficulty in breathing for two days. The patient expired after 15 days of hospitalization.

**Fig. 1.**
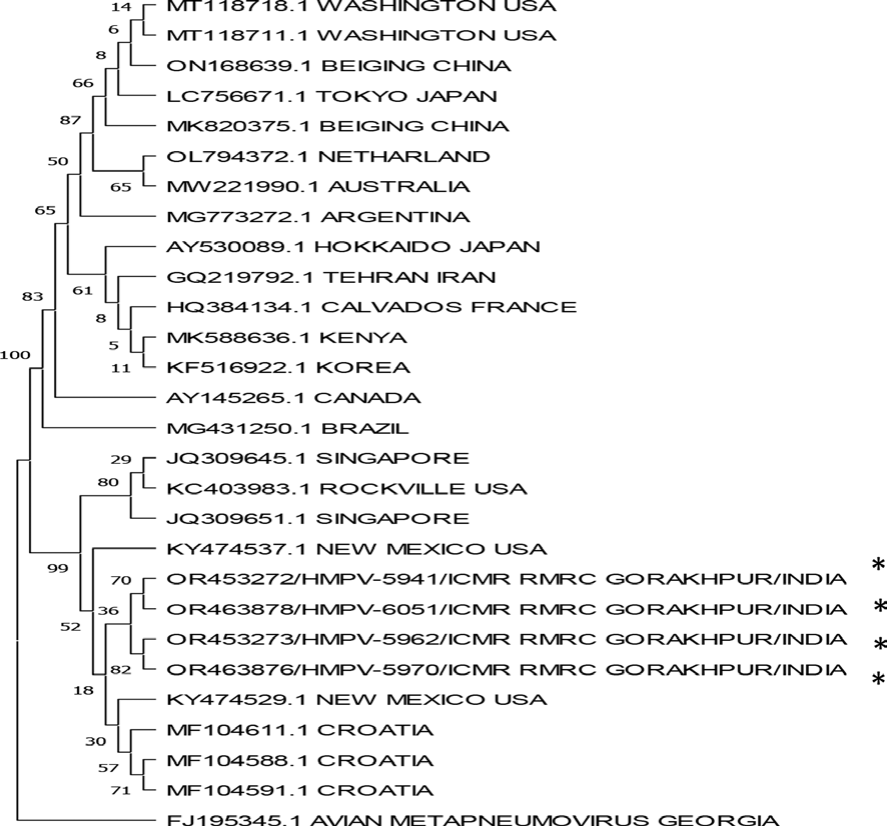
Genetic relatedness between the matrix (M) protein gene of human metapneumovirus (hMPV). The sample strains are indicated by an asterisk (*) while others are reference strains.

The above four nasal and throat swab samples were PCR amplified and the products were processed for Sanger sequencing. The sequences show close resemblance with the clade of Singapore and USA hMPV isolates, clearly showing the detection of hMPV virus in paediatric population of eastern Uttar Pradesh, India (Fig. 1). The PCR amplified product of Matrix gene sequence used in the phylogenetic analysis has been deposited in GenBank under accession no. OR453272/OR453273/OR463876/OR463878.

## Discussion

hMPV has been detected globally since its discovery in 2001 [17]. It is one of the causative agents for respiratory infection, especially in newborns and children. In the present study, hMPV was detected in 4% (04/100) of the ARI cases. This was slightly higher than a study [18] in which the samples positive for hMPV was 3% however the sample size in our study was lower than Banerjee *et al*. According to another study from Lucknow, 188 Nasopharyngeal aspirates (NPAs) were collected from children aged 0–14 years who were hospitalized with signs and symptoms of ALRI (acute lower respiratory infections) and were screened for respiratory viruses. In their study, hMPV was detected in 1.1% of the cases [19], which is also lower than the present study. Some other studies from India also shows hMPV positivity rate: 4% in Chennai, 5% in Pondicherry, 3.6% in Lucknow and 3% in Kolkata [6,20,22]. However, in the above studies from India the age criteria of the children were different than in our study. There is very low reporting of hMPV in ARI cases in children below 5 years old from India. The present study is the first study from Gorakhpur region of eastern Uttar Pradesh which determines the prevalence of hMPV in ARI cases.

Respiratory infection is very common among young children. hMPV is one of the leading causes of ARI in children. Despite this, there is lack of information about hMPV from the eastern region of Uttar Pradesh. hMPV presumed to contribute significantly to bronchiolitis and pneumonia in this population, as it has in other populations [23,25]. In our study, in some cases hMPV were significantly associated with pneumonia as well as difficulty in breathing and wheezing which is an indicator of severe pneumonia in young children.

Phylogenetic analysis of the samples, showed close resemblance of hMPV isolates with USA and Singapore clades (Fig. 1). In our study M-protein gene was amplified and sequenced but in most of the study, F and N gene has been used for phylogenetic analysis and lineage detection [18,26]. Though the lineage determination could not be done in our study but in future, determination of hMPV lineage may prove to be important to determine the lineage prevalent in this region.

In a recent study from neighbouring Pakistan, the positivity rate of hMPV was found to be 16.5% which is significantly higher than the present study [27]. As per the reports from China and Japan, frequent transmissions of hMPV strains has been observed with the seeding of new variants which indicated the risk of an hMPV epidemic in those regions [28]. According to surveillance data from the CDC’s National Respiratory and Enteric Virus Surveillance System (NREVSS), hMPV is most active in temperate areas in the late winter and early spring, our study is also concurrent of surveillance data of NREVSS. As India has a diverse climatic condition, varying regions of India may have different hMPV seasonality.

In the present study, the 96 patients who were tested negative for all the possible causative pathogens were enrolled from the Paediatrics department of BRD Medical College, Gorakhpur. It is a tertiary care centre for Eastern Uttar Pradesh, India. The reason for testing negative for possible causative pathogens may be due to end of the window period before their OPD visits or IPD admission. Another reason might be due to some other conditions like bronchial asthma, foreign body aspiration, congenital heart disease, congestive cardiac failure, idiopathic pneumothorax, acute respiratory distress syndrome, trauma and pneumothorax or kidney failure with acidosis or may be the patients have undergone prior antibiotic treatment.

Our study emphasises the significance of hMPV as aetiology of acute respiratory infection (ARI) in children and highlights the necessity of frequent laboratory testing for this virus. More detailed knowledge on the prevalence of hMPV in this region is required.

As this was the pilot study, only 100 samples were collected and tested. In this study, identification and association of hMPV with SARI cases was demonstrated. The determination of the different lineages should also be done for better correlation with SARI cases and case management in future.

## Conclusion

Human metapneumo virus is an important respiratory viral pathogen in the Eastern UP, India, especially in newborns and children. In our study, hMPV has been detected in 4% (04/100) of the ARI cases. The phylogenetic analysis of hMPV showed the close resemblance with the clade of Singapore and USA hMPV isolates. The study clearly indicated the circulation of hMPV virus in eastern Uttar Pradesh in children under the 5 years of age. This study underlines and highlights the necessity of continued surveillance with more number of samples covering all seasons.

## supplementary material

10.1099/acmi.0.000829.v4 Table S1.

## Abbreviations

ARI: acute respiratory infection
hMPV: human metapneumovirus
ICU: intensive care unit
IPD: indoor patient department
OPD: outdoor patient department
SARI: severe acute respiratory infection
WHO: World Health Organization
