# Supplementary material for: Human metapneumovirus (hMPV): an associated etiology of severe acute respiratory infection in children of Eastern Uttar Pradesh, India
Source: Access Microbiol. 2024 Sep 12;6(9):000829.v4. doi: 10.1099/acmi.0.000829.v4 (PMC11391948; doi:10.1099/acmi.0.000829.v4)
Supplement: Table S1. [file acmi-6-00829-s001.pdf]

Supplementary Table S1: The clinical and biochemical findings of hMPV positive patients.

	Patient 1	Patient 2	Patient 3	Patient 4
**Sex**	Male	Male	Female	Female
**Age in months**	24	60	5	44
**Clinical Findings**				
**Body temperature**	Febrile	Febrile	Febrile	Febrile
**Pulse rate**	164/min	NA	98/min	NA
**Respiratory rate**	60/min	NA	52/min	NA
**SPO2**	90%	NA	86%	NA
**Cough**	Yes	Yes	Yes	Yes
**Cold**	Yes	Yes	Yes	Yes
**Vomiting**	4 Day	12 hrs.	2 hrs.	2 Days
**Difficulty in breathing**	1day	12 hrs.	2 hrs.	2days
**Haemoglobin (Hb)**	9.7	10.9	16.1	NA
**TLC**	19200	10200	23500	NA
**PLT**	309000	258000	576000	NA
**Absolute Neutrophil Count**	13.9	NA	NA	15
**ESR**	24	26	NA	NA
**Total Bilirubin**	0.34	0.30	0.75	NA
**Direct Bilirubin**	0.22	0.15	0.42	NA

**Indirect Bilirubin**	0.12	0.15	0.33	NA
**SGOT**	21.6	28.46	46.34	NA
**SGPT**	19.1	17.36	32.95	NA
**S. ALP**	269.6	NA	486.6	NA
**S.Urea**	26.70	22.56	19.86	NA
**S. Creatinine**	0.66	0.64	0.65	NA
**PT/INR**	21	18.7/1.37	NA	NA
**Electrolytes (Na/K/Ca)**	NA	13.9/4.22/1.15	134.4/3.32/1.07	NA
**Typhoid Dot**	Negative	Negative	Negative	NA
**Hospitalisation Days**	3 days	2 days	2 days	15 days
**Outcome**	Discharged	Discharge	Referred to higher centre	Expired

NA: Not Available
